# Understanding of latent tuberculosis, its treatment and treatment side effects in immigrant and refugee patients

**DOI:** 10.1186/1756-0500-6-342

**Published:** 2013-08-29

**Authors:** Katie Butcher, Beverley-Ann Biggs, Karin Leder, Chris Lemoh, Daniel O’Brien, Caroline Marshall

**Affiliations:** 1 Department of Medicine, University of Melbourne, Grattan St Parkville, Victoria 3050, Australia; 2 Victorian Infectious Diseases Service, Royal Melbourne Hospital, Grattan St Parkville, Victoria 3050, Australia; 3 Department of Epidemiology and Preventive Medicine, Monash University, 99 Commercial Road, Melbourne, Victoria 3000, Australia; 4 Department of Infectious Diseases, Geelong Hospital, Bellerine Street, Geelong, Victoria 3220, Australia

**Keywords:** Latent tuberculosis infection, Health literacy, Immigrant, Refugee

## Abstract

**Background:**

Isoniazid treatment of latent tuberculosis infection (LTBI) is commonly prescribed in refugees and immigrants. We aimed to assess understanding of information provided about LTBI, its treatment and potential side effects.

**Methods:**

A questionnaire was administered in clinics at a tertiary hospital. Total Knowledge (TKS) and Total Side Effect Scores (TSES) were derived. Logistic regression analyses were employed to correlate socio-demographic factors with knowledge.

**Results:**

Fifty-two participants were recruited, 20 at isoniazid commencement and 32 already on isoniazid. The average TKS were 5.04/9 and 6.23/9 respectively and were significantly associated with interpreter use. Approximately half did not know how tuberculosis was transmitted. The average TSES were 5.0/7 and 3.5/7 respectively, but were not influenced by socio-demographic factors.

**Conclusions:**

There was suboptimal knowledge about LTBI. Improvements in health messages delivered via interpreters and additional methods of distributing information need to be developed for this patient population.

## Background

The global epidemiology of tuberculosis (TB) is significantly influenced by human migration. Immigrants from TB endemic nations continue to have TB rates that mirror their country of origin and account for a large proportion of active TB cases in industrialised nations [1-7]. In Australia the incidence of TB in 2009 was 6.4 per 100,000 people [8]. There is incomplete information about the rate amongst refugees and immigrants from high prevalence nations but studies have shown this to be higher than the general Australian population [4,9,10].

The majority of *Mycobacterium tuberculosis* infections do not result in active disease. In greater than 90% of cases the individual remains asymptomatic and non-infectious, with so-called latent tuberculosis infection (LTBI) [11-14]. LTBI may progress to active disease and therefore potentially poses a major public health problem, especially in individuals infected with HIV. LTBI acquired prior to migration may reactivate after settlement in a new country. Effective diagnosis and treatment of LTBI are integral to TB prevention and control in developed nations. The American Thoracic Society and the Centers for Disease Control and Prevention recommends several regimens for treatment of LTBI. The regimen most commonly used in Australia is 300 mg isoniazid once daily for nine months, which reduces the risk of active disease by approximately 80% [15]. Other less commonly used options include shorter, rifampicin-containing regimens. Isoniazid has been linked with serious side effects, the most severe being hepatotoxicity, so careful monitoring is needed [15,16]. Adherence may be variable, largely due to the lengthy treatment duration, lack of disease symptoms and potential adverse events [17-20]. It is thereby essential that patients on isoniazid are thoroughly informed about the treatment benefits, the importance of strict adherence to the medication regimen and the symptoms that may indicate serious side effects.

Immigrant and refugee patients often have low health literacy, posing challenges for health education [14]. Many refugees come from low socio-economic, non-English speaking backgrounds or have limited education and poor knowledge of health and health services. Inadequate health literacy along with language barriers and different cultural and religious beliefs have been associated with lower adherence levels amongst these patients on isoniazid for LTBI [13,18,19,21-24].

In Victoria, patients requiring assessment and management of LTBI are usually referred by their general practitioner to infectious diseases clinics at tertiary hospitals. The Victorian Infectious Diseases Service (VIDS) at the Royal Melbourne Hospital (RMH) assesses large numbers of immigrants and refugees with active TB and LTBI at two clinics, the immigrant and refugee clinic and the TB clinic. Those attending the immigrant and refugee clinic are usually refugees or asylum seekers, who are often recently arrived and may have several medical problems. The patients attending the TB clinic tend to have a single problem and are more likely to be migrants. Currently, treating physicians educate patients about LTBI, isoniazid treatment and possible side effects. However, it is not known to what extent patients understand or remember information they have been given and whether this is influenced by education, ethnicity or gender. Anecdotal reports suggest that patients sometimes take medication incorrectly and forget important potential side effects. The objectives of this study were firstly, to assess refugee and immigrant patients’ knowledge and understanding of information given about LTBI, the treatment regimen and potential side effects of isoniazid and secondly, to evaluate whether demographic factors were associated with knowledge and understanding in order to determine if better methods of providing information are required.

## Methods

### Study design and data collection

RMH is a university-affiliated, tertiary referral hospital. A convenience sample of patients attending the immigrant/refugee and the TB clinics between May 5th and October 9th 2009 was recruited, consisting of patients referred specifically because of a positive QuantiFERON®-TB Gold (Cellestis Ltd, Carnegie, Victoria, Australia) or tuberculin skin test (1 ml of a 100 IU PPD solution [CSL] injected intra-dermally). We intended to recruit at least 50 participants, based on what we perceived would give a reasonable indication of patient knowledge and what was feasible given the available personnel and time frame.

Patients diagnosed with LTBI were included if they were commencing or already taking isoniazid therapy for LTBI, were born in a country with a high prevalence of TB and had lived in Australia for less than ten years. We reviewed all histories of patients booked into each clinic to try to capture all potential participants. Approximately 55 patients newly commenced isoniazid during the study period, although we do not have denominator figures for those already on isoniazid. We did not favor or exclude on specific country of birth or language criteria, but there needed to be clinic space and both interviewer and interpreter time available after (See Additional file 1) or before (See Additional file 2) the patient appointment in order to make it feasible to conduct the interview.

Usual clinical management of LTBI at VIDS includes a detailed verbal explanation about LTBI and the isoniazid treatment regimen given by the treating physician at the initial consultation prior to commencement of treatment. Interpreters are used at every visit where necessary (including follow-up visits), although occasionally this may be via the telephone interpreter service. On most occasions, qualified interpreters are used, but there is no requirement for them to be qualified in medical terminology. Rarely, family members or friends are used as interpreters if there is no trained interpreter available. Commencement of isoniazid therapy would usually be deferred if there is no suitable interpreter available.

Written information is available in English and some of the other languages spoken by our patients, but is not always given to patients. The potential side effects of isoniazid are verbally described by the doctor, as is the course of action to take if these occur. Patients opting for treatment are usually given nine months of daily isoniazid. Alternative rifampicin-containing regimens are rarely used in our clinic. Pyridoxine is sometimes also prescribed [15]. Patients over the age of 35 are not usually offered isoniazid therapy because of the potential for age related hepatotoxicity [15]. Patients on isoniazid are always assessed after one month of treatment and then at one to three month intervals over the treatment course, depending on other co-morbidities and perceived need by treating physician. Liver function is tested at baseline but not routinely thereafter, unless the patient reports symptoms suggestive of hepatotoxicity. Education would usually be given by the treating physician at each visit depending on perceived need.

Two questionnaires were designed specifically for this study (See Additional file 1 and Additional file 2). We found no other published questionnaires covering areas that were of interest to the authors and none that had been administered to refugee patients. We pilot tested the questionnaires on nine patients over a two week period, resulting only in minor changes to the structure of the questionnaires, so all data from the pilot was included in the analysis.

Questionnaire 1 contained 23 questions consisting of eight socio-demographic questions and 15 to assess knowledge and understanding of LTBI, isoniazid regimen and side effects. It was administered to patients at the initial consultation when isoniazid treatment was first prescribed. The questionnaire was completed following the consultation to assess immediate recall of information provided by the doctor.

Questionnaire 2 consisted of the same questions as Questionnaire 1 with three additional questions regarding the number of missed days of isoniazid and whether the diagnosis had affected either the patient or their family’s daily lives. It was administered to assess long term recall at a follow-up consultation after participants had been taking isoniazid for at least one month. Questionnaire 2 was administered prior to the patient seeing the doctor on these follow-up visits. Where possible, participants completed both Questionnaire 1 and Questionnaire 2. Questionnaires were read aloud to participants, through the use of an interpreter if necessary.

### Data analysis

A “Total Knowledge Score” (TKS) was calculated for each participant by adding the score from five questions that were deemed (by consensus from the physicians in the clinic) to be the best indicators of overall LTBI knowledge, the treatment regimen and isoniazid side effects (including elements from questions 9, 10, 15, 19 and 20 see Additional file 2). Participants were awarded two points for knowing they had LTBI and one point for a reference to TB. Two points were given if the participant mentioned they were taking the same medication as prescribed and one point if they didn’t distinguish between INH and pyridoxine. Knowing TB was spread via some form of microbe, that nausea/vomiting, abdominal pain or jaundice were side effects of INH and mentioning to stop medication if side effects occurred were awarded one point each giving a Total Knowledge Score out of nine. Binary TKS categories of high (>6) and low (≤6) were created as the outcome variable for regression models. Higher TKS indicated better results. Predictors of high TKS score were analysed using univariable and multivariable Poisson regression with robust standard errors. A Poisson approach was preferred over logistic modeling secondary to the relative frequency of the outcome variable (high TKS score was recorded for 48% of the sample). Poisson modeling also permitted direct calculation of relative risk whereas a logistic approach, in light of the frequency, would likely have returned over-estimates of relative risk. Candidate predictors for inclusion in the multivariable model were identified as those considered to be clinically important by the researchers coupled with performance on univariable modeling. The number of outcomes (n = 22) limited adjusted modeling to a maximum of two concurrent explanatory variables to avoid over-fitting of the data. Collinearity between predictor variables was tested for. Interactions between predictors, as distinct from collinearity were examined using a likelihood-ratio test. Overall goodness of fit of the Poisson models was assessed using a Poisson chi-square goodness of fit test. Competing multivariable Poisson model solutions were compared using a bias-corrected Akaike Information Criterion [25] to identify the most parsimonious combination of predictor variables that was consistent with clinical expectations.

A “Total Side Effect Score” (TSES) was also generated by awarding one point for each of the seven potential side effects of isoniazid remembered by the participant (liver problems, nausea/vomiting, abdominal pain, jaundice, nerve problems, tingling/numbness in extremities and rash). Higher TSES indicated better results.

Data were analyzed using Microsoft Excel™ 2003 and STATA version 10 (StataCorp, College Station, TX). Results were considered statistically significant if the p-value was <0.05 and all reported confidence intervals are at 95%.

### Ethics approval

This study was approved by the Melbourne Health Human Research Ethics Committee Quality Assurance Stream. Verbal consent was obtained from all participants.

## Results

### Profile of study participants

Fifty-three patients were asked to take part in the study, of whom 52 agreed to participate. Thirty-two had already commenced isoniazid treatment and completed Ques1tionnaire 2 at a follow-up consultation. Twenty participants started on isoniazid during the study and completed Questionnaire 1 at their initial consultation. Of these, 14 completed Questionnaire 2 at a subsequent consultation, giving a total of 46 participants completing Questionnaire 2. Therefore, 20 patients completed Questionnaire 1, 46 completed Questionnaire 2 and 14 completed both.

Table 1 shows the socio-demographic characteristics of study participants. Participants came from 18 different countries (most commonly India, Ethiopia and Burma) and spoke 16 different primary languages (most commonly English and Nepalese). Twenty-two patients required interpreters, who were available for all consultations. Languages included Amharic, Arabic, Burmese, Chin Hakka, Hindi, Karen, Korean, Oromo, Swahili, Somali and Vietnamese. Questionnaires were translated directly to the participants. Fifteen participants were born in sub-Saharan Africa, 17 in Asia, 18 in the Indian sub-continent and 2 in the Middle East. Thirty-two participants (61.5%) had completed tertiary education, while two had no formal education at all.

**Table 1:** Socio-demographic characteristics of study participants

	**Tertiary educated**	**Not tertiary educated**	**Total**
**Population characteristics**	**(N = 32)**	**(N = 20)**	**(N = 52 **^**a**^**)**
	***n *(%)**	***n *(%)**	***n *(%)**
Clinic			
Refugee & Immigrant	4 (21.0)	15 (79.0)	19 (36.5)
TB	28 (84.9)	5 (15.1)	33 (63.5)
Gender			
Male	14 (60.9)	9 (39.1)	23 (44.2)
Female	18 (62.1)	11 (37.9)	29 (55.7)
Interpreter Required			
Yes	6 (27.3)	16 (72.7)	22 (42.3)
No	26 (86.7)	4 (13.3)	30 (57.7)
Reads English			
Yes	32 (71.1)	13 (28.9)	45 (86.5)
No	0	7 (100)	7 (13.5)
Lived in a Refugee Camp			
Yes	2 (20.0)	8 (80.0)	10 (19.2)
No	30 (71.4)	12 (28.6)	42 (80.7)
Time in Australia			
< 6 months	2 (28.6)	5 (71.4)	7 (13.5)
6 months- < 1 year	11 (57.9)	8 (42.1)	19 (36.4)
1- < 5 years	15 (68.2)	7 (31.8)	22 (42.3)
5-10 years	4 (100.0)	0	4 (7.7)
Born in sub-Saharan Africa			
Yes	5 (33.3)	10 (66.7)	15 (28.8)
No	27 (72.9)	10 (27.1)	37 (71.2)
Age (years)			
Mean	28.0	27.9	27.9
Median	28	28	27.5
Range	18-37	18-36	18-37

^a^ Total number of patients in study.

### Participant understanding of latent tuberculosis and treatment

The average TKS for Questionnaire 1 was 5.04 ± 2.50 (range 1–8) out of a total of nine. The average TKS for Questionnaire 2 was 6.23 ± 1.80 (range 3–9). Only three participants got the maximum score of nine (15%). Seventy-five per cent (n = 15) of those who completed Questionnaire 1 and 89% (n = 41) of participants who completed Questionnaire 2 understood they had LTBI. Typical correct responses were “non-active TB”, “non-serious TB” or “sleeping TB”. Responses to both Questionnaires 1 and 2 showed over 90% of participants knew that LTBI was asymptomatic and non-transmissible (Table 2). There was a range of misconceptions about how TB was spread including via water, dust, wind or pollution.

**Table 2:** Participant understanding of LTBI and isoniazid treatment

	**Questionnaire 1**	**Questionnaire 2**
	**(N = 20 **^**a**^**)**	**(N = 46 **^**b**^**)**
**Understanding of LTBI and isoniazid treatment**	***n *(%)**	***n *(%)**
Why are you coming to this clinic?		
Knew they had LTBI	15 (75.0)	41 (89.1)
Couldn’t distinguish between TB & LTBI	5 (25.0)	4 (8.7)
Unsure	0 (0.0)	1 (2.2)
What is TB caused by?		
Knew it was caused by a transmissible microbe	9 (45.0)	22 (47.8)
Unsure	11 (55.0)	24 (52.2)
Does this condition make you sick?		
Yes	0 (0.0)	2 (4.4)
No	20 (100.0)	44 (95.7)
Can you give this condition to anyone else?		
Yes	1 (5.0)	1 (2.3)
No	18 (90.0)	43 (93.5)
Unsure	1 (5.0)	2 (4.4)
How will taking the tablets help you?		
Reduce the risk of active TB in the future	19 (95.0)	41 (89.1)
Unsure	1 (5.0)	5 (10.9)
How many tablets will/do you take a day?		
Answer same as medication prescribed	14 (70.0)	42 (91.3)
Answer different to medication prescribed	6 (30.0)	4 (8.7)
Do you know the name of the tablets?		
Yes	4 (20.0)	15 (32.6)
No	16 (80.0)	31 (67.4)
How many times a day will/do you take the tablets?		
Once	16 (80.0)	45 (97.8)
Three Times	0 (0.0)	1 (2.2)
Unsure	4 (20.0)	0 (0.0)
How long will you need to take the tablets for?		
9 months	16 (80.0)	35 (76.1)
6–9 months	4 (20.0)	7 (15.2)
6 months	0 (0.0)	3 (6.5)
Unsure	0 (0.0)	1 (2.2)
How many days of tablets have you missed in the last months?		
None	N/A	25 (54.3)
1–5 days	N/A	17 (37.0)
5–10 days	N/A	4 (8.7)

LTBI = Latent Tuberculosis Infection; TB = Tuberculosis.

^a^ 20 patients started on isoniazid and completed Questionnaire 1.

^b^ 46 patients completed Questionnaire 2.

There was good understanding of the treatment regimen; the reported medication dose matched that prescribed in 91.3% (n = 42) of participants who completed Questionnaire 2. However, 37.0% (n = 17) of participants stated they had missed 1–5 days of medication in the past month, with “forgetting” being the most common reason (Table 2).

### Participant understanding of isoniazid side effects

The mean TSES for participants completing Questionnaires 1 and 2 was 5.0 ± 1.8 (range 2–7) and 3.5 ± 1.8 (range 0–7) respectively. Only two participants who completed Questionnaire 2 could mention all seven side effects and two could not recall any (Table 3). Participants also proffered other side effects not included in the score, such as dizziness, change in urine colour, night sweats, acne and hair loss. All 46 participants who completed Questionnaire 2 said they would contact a doctor should they experience side effects and half said they would stop taking isoniazid (n = 24, 52.2%).

**Table 3:** Potential isoniazid side effects reported by participants who completed questionnaire 2

	**Participant responses**	
	**(N = 46 **^**a**^**)**	
	**Yes**	**No**
**Isoniazid side effect**	***n *(%)**	***n *(%)**
Liver Problems	25 (54.3)	21 (45.7)
Nausea/ Vomiting	25 (54.3)	21 (45.7)
Abdominal Pain	26 (56.5)	20 (43.5)
Jaundice	21 (45.7)	25 (54.3)
Nerve Problems	19 (41.3)	27 (58.7)
Tingling/Numbness in extremities	24 (52.2)	22 (47.8)
Rash	23 (50.0)	23 (50.0)

^a^ 46 participants completed Questionnaire 2.

### Effect of socio-demographic determinants on participant understanding

Results of the univariable and multivariable analyses showing predictor variables for TKS are shown in Table 4. Having an interpreter during the consultation was a significant predictor of a low TKS, in both univariable and multivariable analysis on Questionnaire 2.

**Table 4:** Predictors of total knowledge score (Questionnaire 2)

	**Total knowledge Score- questionnaire 2**							
	**Univariable analysis**					**Multivariable analysis**		
	**High score** ^**a**^	**Low score** ^**b**^						
	**N = 22**	**N = 24**						
**Variables**	**( *n *)**	**( *n *)**	**uIRR**	**(95% CI)**	***P***	**aIRR**	**(95% CI)**	***P***
Age, years	22	24	1.02	(0.89-1.75)	0.737			
Interpreter	6	15	0.23	(0.07-0.79)	0.019	0.313	(0.14-0.69)	0.004
I & R Clinic	7	10	0.66	(0.20-2.19)	0.490	1.75	(0.93-3.30)	0.083
Male sex	9	12	0.69	(0.22-2.23)	0.537			
Tertiary educated	16	13	2.26	(0.66-7.76)	0.197			
Lived in a refugee camp	3	6	0.47	(0.10-2.18)	0.338			
Mean number of clinic visits	2.5	2.6	5.53	0.59-51.64)	0.134			
Residence in Australia > 1 year	13	12	1.44	(0.45-4.64)	0.537			
Sub-Saharan African country of birth	7	6	1.13	(0.32-3.98)	0.845			

I & R Clinic = Immigrant and Refugee Clinic; CI = Confidence Interval; uIRR = unadjusted Incidence Rate Ratio; aIRR = adjusted Incidence Rate Ratio.

^a^ High Score, greater than 6/9.

^b^ Low Score, less than or equal to 6/9.

## Discussion

This is the first Australian study to provide a snapshot of the level of understanding about LTBI, isoniazid treatment and its side effects amongst refugee and immigrant patients attending a tertiary referral hospital. The study identified significant gaps with almost half not knowing how TB was spread and many not able to recall the side effects of isoniazid. The study identified an association between the use of an interpreter and having a low overall knowledge score. These findings demonstrate a critical need for improved strategies for the provision of health education to immigrant and refugee patients receiving treatment for latent tuberculosis to ensure its effectiveness.

Many misconceptions about the way TB is spread were reported. This finding is consistent with other studies in different ethnic groups, indicating that cultural beliefs may have a confounding influence on disease knowledge [14,24,26]. Furthermore, misconceptions about the spread of TB have previously been shown to impact upon an individual’s willingness to take prophylactic medication and compliance with isoniazid [19,21,23,26].

The incomplete understanding of isoniazid side effects is similar to that found amongst Latino immigrants to the US [14]. Limited recall of the potential side effects of isoniazid amongst participants is concerning, most significantly because of the hepatotoxicity risk. Immigrant and refugee patients from non-English speaking backgrounds may have particular trouble understanding the complex side effects of isoniazid, especially if they have had limited education about health. Medical terms for these side effects may not translate specifically into other languages and the meaning may be difficult to convey.

Having an interpreter was significantly associated with a 69% lower chance of having a high score after adjustment for clinic day. This finding has not been described before in similar studies. It may indicate inadequate training of interpreters with either lack of knowledge of particular terms used in discussion of LTBI or absence of such words in some languages, making direct translation impossible. Another possible explanation is that the need for an interpreter is a marker for major differences in understanding of health, illness and healing that go beyond the capacity of an interpreter to deal with. Alternatively, English-speaking clinicians in a majority English-speaking culture may not communicate well with patients who need interpreters.

Nearly 46% of patients reported missing treatment for between one and ten days in the prior month, with most reporting that they had forgotten to take their tablets. This is a concerning figure with potential impacts on the effectiveness of treatment. Whilst health education activities may not improve memory, repeated explanations about the potential consequences of forgetting may help to crystallize the importance of good adherence. Frequent clinic visits, for example monthly, may also help to reinforce these messages and improve adherence. Newer treatment regimens, such as weekly isoniazid and rifapentine for 12 weeks, are simpler, may improve adherence and may be suitable for these patients [27]. This regimen was not available in Australia at the time of the study.

The study has several potential limitations. The number of participants was relatively small. Despite this, there were still some significant results found in the multivariable regression. Although a standardised explanation was not provided by physicians, a similar way of explaining concepts is used by the doctors in the clinics and there is very little turn-over of doctors. We cannot be certain, however, whether all concepts tested on the questionnaires were explained to all patients. There may also have been a loss of information accuracy through the use of an interpreter. Furthermore, baseline knowledge of participants was not tested. The questionnaire utilised was developed specifically for the purpose of this study and has not been used within other population groups. Whilst it has face validity, it may need to be validated in other studies.

## Conclusions

This study found that refugee and immigrant patients attending two infectious diseases outpatient clinics at a tertiary referral hospital had a suboptimal level of understanding and knowledge of latent TB, its treatment regimen and treatment side effects. These findings have significant implications for clinical practice. The association between having an interpreter and low knowledge and understanding is particularly worrying as it suggests that patients requiring an interpreter may be at a disadvantage, for which use of an interpreter is insufficient to compensate. Medical education programs are needed to improve interpreters’ knowledge about latent TB (and other diseases) in order to transfer accurate health messages to patients. There is currently no specialist training for interpreters in medical vocabulary and no requirement that there should be such training. Any training that takes place is through self-funded personal development. Outsourcing of interpreting services may prevent the accumulation of organisational experience in the field or in specific institutions. One possible avenue to address this deficit is offering certified short courses for interpreters who work in health care settings. Similarly, immigrant and refugee populations would benefit from linguistically and culturally appropriate health education. This would be particularly beneficial for refugee patients from non-English speaking backgrounds who may have low health literacy and limited access to accurate information. Information is available in some languages other than English, but not in the languages most commonly spoken by our patients. Translation is limited by lack of resources. Another, more complex response would be the development of a bilingual health worker role to provide support and education to individual patients and ethnic communities beyond simple interpreting. Findings from this study could help to inform providers of TB care how best to educate and motivate patients from very different cultural and linguistic backgrounds about the importance of adherence to treatment for latent TB.

## Competing interests

The authors declare that they have no competing interests.

## Authors’ contributions

KB participated in design of the study, collected and analysed the data and drafted the manuscript. BAB assisted in study conception and design, oversaw data collection and analysis and helped draft the manuscript. KL assisted in study conception and design, oversaw data collection and analysis and helped draft the manuscript. CL oversaw data collection and analysis and helped draft the manuscript. DO oversaw data collection and analysis and helped draft the manuscript. CM assisted in study conception and design and coordination, oversaw data collection and analysis and helped draft the manuscript. All authors read and approved the final manuscript.

## Authors’ information

KB was an honours science student in the Department of Medicine at the University of Melbourne at the time of this study.

BAB is an infectious diseases physician at the Royal Melbourne Hospital and Professor at the Department of Medicine, University of Melbourne.

KL is an infectious diseases physician at the Royal Melbourne Hospital and Associate Professor at the Department of Epidemiology and Preventive Medicine, Monash University.

CL was an infectious diseases physician at the Royal Melbourne Hospital and PhD student at the Department of Medicine, University of Melbourne at the time of this study.

DO is an infectious diseases physician at the Royal Melbourne Hospital and Geelong Hospital.

CM is an infectious diseases physician at the Royal Melbourne Hospital and Associate Professor at the Department of Medicine, University of Melbourne.

## Supplementary Material

Additional file 1 **Survey instrument.** This file contains Questionnaire 1 administered to participants. Key sections have been reported in this paper. Click here for file

Additional file 2 **Survey instrument.** This file contains Questionnaire 2 administered to participants. Key sections have been reported in this paper. Click here for file
