# Supplementary material for: Understanding of latent tuberculosis, its treatment and treatment side effects in immigrant and refugee patients
Source: BMC Res Notes. 2013 Aug 29;6:342. doi: 10.1186/1756-0500-6-342 (PMC3766130; doi:10.1186/1756-0500-6-342)
Supplement: Additional file 1 — Survey instrument. This file contains Questionnaire 1 administered to participants. Key sections have been reported in this paper. [file 1756-0500-6-342-S1.doc]

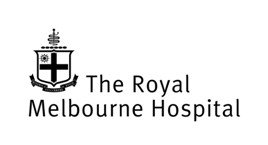


**QA Number- QA2009025**

**Date of Consultation- Day of Consultation-** Tuesday or Friday

Patient Study Number- Doctor’s Study Number- Gender-

Doctor’s Name- Interpreter- Y or N

**PATIENT UNDERSTANDING OF LATENT TB, ITS TREATMENT AND SIDE EFFECTS**

**Questionnaire One- Instant recall (New patients)**

**Explanation about study to patient:**

We are asking patients some questions about what they have been told by the doctor about why they are coming to the clinic and their treatment. This is called an audit and we hope to use the answers that you give us to improve how we explain things to patients in the future. Some of the questions that we are asking are part of the normal consultation and some are questions that we would not normally ask you. It will only take a few minutes to answer these questions. You do not have to answer the questions if you do not wish, but we would appreciate it if you would. We will keep the answers to your questions but will not show them to anyone else.

**Verbal consent to participate in audit obtained:**

Yes 🞎 No 🞎

**PATIENT UNDERSTANDING OF LATENT TB, ITS TREATMENT AND SIDE EFFECTS**

**Questionnaire One- Instant recall (New patients)**

** **

1. **What is your preferred language?**........................................................................................
2. **Can you read in your preferred language?** Yes 🞎 No 🞎

**Or/and can you read in English?** Yes 🞎 No 🞎

Unsure- 🞎

Key comments/Quotes………………………………………………………..........................

1. **If unable to read English-**

**Do you have a family member or friend that can read in your preferred language?**

Yes 🞎 No 🞎

**Or/and do you have a family member or friend that can read in English?**

Yes 🞎 No 🞎

Unsure- 🞎

Key comments/Quotes………………………………………………………………………..

1. **Where were you born?**...........................................................................................................
2. **How long have you lived in Australia for?**

Exact length of time-…………………………………………………………………………

Less than a year 🞎

One-Five years 🞎

Five-Ten years 🞎

More than 10 years 🞎

Was born here 🞎

Unsure 🞎

Other……………………………………………………………………………………….....

Key comments/Quotes………………………………………………………………………..

1. **Have you ever lived in a Refugee Camp?** Yes 🞎 No 🞎

Unsure- 🞎

Other………………………………………………………………………….........................

Key comments/Quotes………………………………………………………………………..

1. **Who do you live with in Australia?**

Immediate Family- Yes 🞎 No 🞎

Extended Family- Yes 🞎 No 🞎

Friends- Yes 🞎 No 🞎

Alone- Yes 🞎 No 🞎

Other………………………………………………………………………………………….

Key comments/Quotes………………………………………………………………………..

1. **How many years of schooling did you do? (Please circle)?**

None 1 2 3 4 5 6 7 8 9 10 11 12 13 Tertiary Education

Unsure- 🞎

Key comments/Quotes………………………………………………………………………..

1. **Why are you coming to this clinic?**

Knows they have a TB but cannot distinguish between active and latent- 🞎

Thinks they have active TB- 🞎

Has a concept of Latent TB- 🞎

Unsure why they are coming to the clinic- 🞎

Other………………………………………………………………………………………….

Key comments/Quotes………………………………………………………………………..

1. **Do you know what this condition (TB) is caused by?**

Has concept of Germ/microbe- Yes 🞎 No 🞎

Unsure what the condition is caused by- 🞎

Other………………………………………………………………………………………….

Key comments/Quotes………………………………………………………………………..

1. **Do you know how the doctor discovered this condition in yourself?**

Tests- 🞎

Blood test- 🞎

Skin test- 🞎

Chest x-ray- 🞎

Unsure of how it was discovered- 🞎

Other………………………………………………………………………….........................

Key comments/Quotes………………………………………………………………………..

1. **How does this condition affect you?**

Understands that the condition is currently not making them sick- Yes 🞎 No 🞎

Unsure of how the condition affects them- 🞎

Other………………………………………………………………………………………….

Key comments/Quotes………………………………………………………………………..

1. **Can you give this condition to anyone else?** Yes 🞎 No 🞎

Unsure- 🞎

Other………………………………………………………………………….........................

Key comments/Quotes………………………………………………………………………..

1. **How will taking the tablets help you?**

Understands that it will reduce their chance of getting sick in the future- Yes 🞎 No 🞎

Unsure of how the tablets will help them- 🞎

Other………………………………………………………………………………………….

Key comments/Quotes………………………………………………………………………..

1. **How many tablets do you need to take a day for this condition?**

Four tablets- 🞎

Three tablets + one vitamin- 🞎

Three tablets- 🞎

***Prompt*** Knew drug names- 🞎

Doesn’t know how many tablets to take- 🞎

Other………………………………………………………………………………………….

Key comments/Quotes………………………………………………………………………..

Medication patient is actually on……………………………………………………………..

1. **How many times a day will you need to take the tablets?**

Once- 🞎

Twice- 🞎

Threes times- 🞎

Unsure- 🞎

Other………………………………………………………………………………………….

Key comments/Quotes………………………………………………………………………..

1. **How long will you need to take the tablets for?**

9 months- Yes 🞎 No 🞎

6 months- Yes 🞎 No 🞎

6-9months- Yes 🞎 No 🞎

Unsure- 🞎

Other………………………………………………………………………………………….

Key comments/Quotes………………………………………………………………………..

Actual length of treatment…………………………………………………………………..

1. **What are the side effects of the tablets that you need to look out for?**

**Before Prompt After Prompt**

Hepatotoxicity (Liver problems) Yes 🞎 No 🞎 Yes 🞎 No 🞎

Nausea/Vomiting- Yes 🞎 No 🞎 Yes 🞎 No 🞎

Abdominal or liver pain- Yes 🞎 No 🞎 Yes 🞎 No 🞎

Jaundice/Yellowing of eyes/skin- Yes 🞎 No 🞎 Yes 🞎 No 🞎

Neurotoxicity (Nerve problems) Yes 🞎 No 🞎 Yes 🞎 No 🞎

Tingling/Numbness in fingers/toes and altered sensation- Yes 🞎 No 🞎 Yes 🞎 No 🞎

Fever (Not mentioned on Friday)- Yes 🞎 No 🞎 Yes 🞎 No 🞎

Rash Yes 🞎 No 🞎 Yes 🞎 No 🞎

Unsure of any side effects- 🞎

Other side effects stated by the patient……………………………………………………….

Key comments/Quotes………………………………………………………………………..

1. **What should you do if you get any of these side effects?**

Stop taking the tablets- Yes 🞎 No 🞎

See Doctor- Yes 🞎 No 🞎

Have a Blood Test- Yes 🞎 No 🞎

Unsure- 🞎

Other………………………………………………………………………………………….

Key comments/Quotes………………………………………………………………………..

1. **Do you know of anyone else on the same TB treatment as you are?** Yes 🞎 No 🞎

Unsure- 🞎

Other………………………………………………………………………….........................

Key comments/Quotes………………………………………………………………………..

1. **Do you think the explanation of latent TB given to you by the doctor was adequate?** Yes 🞎 No 🞎

Unsure- 🞎

Other………………………………………………………………………….........................

Key comments/Quotes………………………………………………………………………..

1. **How would you like to receive information about latent TB?**

**After prompt**

Nurse- Yes 🞎 No 🞎

Pamphlet in English- Yes 🞎 No 🞎

Pamphlet in own language- Yes 🞎 No 🞎

Video/DVD- Yes 🞎 No 🞎

Unsure- Yes 🞎 No 🞎

Other……………………………………………………………………………………………...

1. **Did you receive this information sheet from the doctor?(show sheet)** Yes 🞎 No 🞎

Unsure-🞎

Patient seen receiving sheet- Yes 🞎 No 🞎
